# The complete chloroplast genome of *Cycas Szechuanensis*, an extremely endangered species

**DOI:** 10.1080/23802359.2018.1507635

**Published:** 2018-09-10

**Authors:** Yaling Wang, Zefu Wang, Lei Zhang, Xiaoyue Yang

**Affiliations:** a College of Life Science and Engineering, Northwest Minzu University, Lanzhou, China;; b State Key Laboratory of Grassland Agro-Ecosystem, College of Life Science, Lanzhou University, Lanzhou, China

**Keywords:** Complete chloroplast genome, *Cycas szechuanensis*, phylogenetic analysis

## Abstract

In this study, we determined the complete chloroplast genome of *Cycas szechuanensis* (Cycadaceae, *Cycas*) using the Illumina sequencing data. The genome is 162,083 bp in length, including a pair of inverted repeats (IRs) of 25,003 bp each, which are separated by a large single-copy region (LSC) and a small single-copy region (SSC) with the size of 88,970 and 23,108 bp, respectively. The genome comprises 131 encoded genes in total, including 86 protein-coding genes (82 PCG species), 8 ribosomal RNA genes (4 rRNA species), and 37 transfer RNA genes (30 tRNA species). The overall GC content of the *C. szechuanensis* chloroplast genome is 39.42%, while the LSC, SSC, and IRs occupy 38.70, 42.03 and 36.52%, respectively. Based on complete chloroplast genome sequences from 13 species, we reconstructed the phylogenetic relationship of the 13 species and found that *C. szechuanensis* is closely related to *C. debaoensis*.

*Cycas szechuanensis*, an endemic species of China, belongs to cycad family (Cycadaceae, *Cycas*). It is listed as Critically Endangered in the latest IUCN red list (http://www.iucnredlist.org/search). Herein, we reported the chloroplast genome of *C. szechuanensis*. The annotated chloroplast genome has been submitted to GenBank under the accession number of MH341576.

The fresh leaves of a single *C. szechuanensis* were collected from Emei Mountain in Sichuan Province, China (29°22′N, 103°17′N). Voucher specimen of the species was stored in the Key Laboratory of Bio-resource and Eco-environment of Ministry of Education (Sichuan, China). The total DNA was extracted from the leaves with the modified CTAB method (Doyle and Doyle 1987). And the Hiseq2500 System was used to perform the whole-genome sequencing (Illumina, USA). About 10G high-quality base pairs of sequencing data in total were obtained. The software NOVOPlasty v2.5.9 (Dierckxsens et al. 2017) was used for genome assembly. Then, the resulting contigs were aligned to the reference *C. revoluta* (NC_020319.1) chloroplast genome with Bwa (Li 2013) and Samtools (Li et al. 2009) and processed into a complete genome by Geneious v 11.1.14 (Kearse et al. 2012). GapCloser (Luo et al. 2012) was used to fill the gaps. Finally, a l62,083-bp chloroplast genome of *C. szechuanensis* was gained. The annotation was dealt with Plann (Huang and Cronk 2015), and Sequin software (NCBI website) was adopted to correct the annotation.

The complete chloroplast genome of *C. szechuanensis* contains a large single-copy (LSC) region with size of 88,970 bp, a pair of inverted repeat (IRA and IRB) regions of 25,003 bp each, and a small single-copy (SSC) region with the lengths of 23,108 bp. *C. szechuanensis* complete chloroplast genome contains 131 genes, including 86 protein-coding genes (82 PCG species), 37 transfer RNA genes (30 tRNA species) and 8 ribosomal RNA genes (4 rRNA species). Most of these genes occurred as a single copy, while 15 gene species occurred as double copies, including all 4 PCG species (*rps12*, *ycf*2, *ndh*B, *rps*7), 7 tRNA species (*trn*H-GUG, *trn*L-CAA, *trn*V-GAC, *trn*I-GAU, *trn*A-UGC, *trn*R-AGC, *trn*N-GUU) and 4 rRNA species (4.5S, 5S, 16S and 23S rRNA). The overall GC content of *C. szechuanensis* chloroplast genome is 39.42%, and the LSC, SSC, and IR regions occupy 38.70, 42.03, and 36.52%, respectively.

We reconstructed a phylogenetic tree of *C. szechuanensis* and other 12 species based on their complete chloroplast genome sequences to infer the phylogenetic position of *C. szechuanensis*. The 13 sequences were aligned with MAFFT v 7.221 (Katoh and Standley 2013). And the maximum likelihood (ML) tree with 100 bootstrap replicates was performed with RaxML v 8.1.24 (Stamatakis 2014). Though *C. szechuanensis* has overlapping distribution areas with *C. panzhihuaensis*, the ML phylogenetic tree strongly showed that it was closely related to *C. debaoensis*, which distributes father away (Figure 1).

**Figure:**
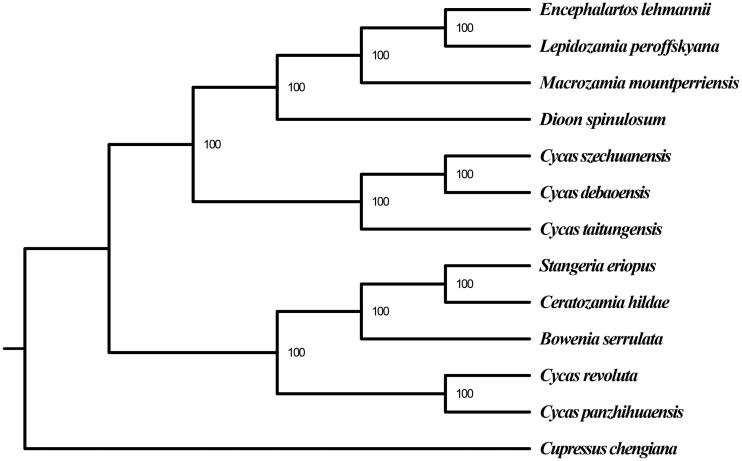
**Figure1.** ML phylogenetic tree based on the complete chloroplast genome sequences of *C. szechuanensis* and other 12 species. Numbers in the nodes are the bootstrap values from 100 replicates. Their accession numbers are as follows: *Encephalartos lehmannii*: NC_027514.1, *Lepidozamia peroffskyana*: NC_027513.1, *Macrozamia mountperriensis*: NC_027511.1; *Dioon spinulosum*: NC_027512.1; *C. debaoensis*: KM459003.1; *C. taitungensis*: NC_009618.1; *Stangeria eriopus*: NC_026041.1; *Ceratozamia hildae*: NC_026037.1; *Bowenia serrulata*: NC_026036.1; *C. revoluta*: NC_020319.1; *C. panzhihuaensis*: NC_031413.1; *Cupressus chengiana*: NC_034788.1.

Above all, this report provides essential data for population, phylogenetic, and other studies of *C. szechuanensis*, as well as Cycadaceae. A good knowledge of its genomic information will help us protect this extremely endangered plant.
